# Circulating immune biomarkers in peripheral blood correlate with clinical outcomes in advanced breast cancer

**DOI:** 10.1038/s41598-021-93838-w

**Published:** 2021-07-13

**Authors:** Natalia Palazón-Carrión, Carlos Jiménez-Cortegana, M. Luisa Sánchez-León, Fernando Henao-Carrasco, Esteban Nogales-Fernández, Massimo Chiesa, Rosalía Caballero, Federico Rojo, María-Adoración Nieto-García, Víctor Sánchez-Margalet, Luis de la Cruz-Merino, the Spanish Breast Cancer Group (GEICAM) and the Spanish Group for Immunobiotherapy of Cancer (GÉTICA)

**Affiliations:** 1 Clinical Oncology Department, Virgen Macarena University Hospital, Seville, Spain; 2 Department of Medical Biochemistry and Molecular Biology, and Immunology, School of Medicine, Virgen Macarena University Hospital, University of Seville, Seville, Spain; 3 GEICAM (Spanish Breast Cancer Research Group), Madrid, Spain; 4 Pathology Department, IIS-Fundación Jimenez Diaz-CIBERONC, Madrid, Spain; 5 Department of Preventive Medicine and Public Health, Faculty of Medicine, University of Seville, Seville, Spain; 6 Medicine Department, University of Seville, Seville, Spain

**Keywords:** Cancer, Immunology, Biomarkers, Oncology

## Abstract

Identification of the different elements intervening at the tumor microenvironment seems key to explain clinical evolution in several tumor types. In this study, a set of immune biomarkers (myeloid derived suppressor cells, regulatory T cells, and OX40 + and PD-1 + T lymphocytes counts) in peripheral blood of patients diagnosed with advanced breast cancer were analyzed along of first line antineoplastic therapy. Subsequently, a comparison between groups with clinical benefit versus progression of disease and with a healthy women cohort was executed. Results reflected that patients showed higher basal levels of myeloid derived suppressor cells (35.43, IR = 180.73 vs 17.53, IR = 16.96 cells/μl; p = 0.001) and regulatory T cells (32.05, IR = 29.84 vs 22.61, IR = 13.57 cells/μl; p = 0.001) in comparison with healthy women. Furthermore, an increase in the number of activated T lymphocytes (expressing OX40), a decrease of immune inhibitory cells (MDSCs and Tregs) and inhibited T lymphocytes (expressing PD-1) were observed along the treatment in patients with clinical benefit (p ≤ 0.001). The opposite trend was observed in the case of disease progression. These findings suggest that some critical immune elements can be easily detected and measured in peripheral blood, which open a new opportunity for translational research, as they seem to be correlated with clinical evolution, at least in ABC.

## Introduction

Breast cancer (BC) is the leading cause of cancer in women with more than 2 million of new cases worldwide every year. At present, although it is considered that 80-85% of the BC patients can be cured, a sizeable proportion recurs in an advanced stage and eventually die as a consequence of the disease^1^.

Immunoediting hypothesis recognizes the crucial role of the different elements of tumor microenvironment (TME), with respect to development and progression of tumors, including breast cancer^2,3^. Consequently, the balance between immune inhibitory and stimulatory signals is determinant to explain the final effect, anergy or activation of the immune system against cancer cells. Density and function of some immune cell populations seem related to the response to treatment and prognosis of cancer^4–7^ and, interestingly, they can be detected and measured not just in TME but even in peripheral blood, what renders many advantages over tissue biopsies^8^. At this point, myeloid derived suppressor cells (MDSCs) and different lymphocyte subpopulations, such as regulatory T cells (Tregs)^4^, activated CD4 + and CD8 + T lymphocytes (expressing OX40) or inhibited T lymphocytes (expressing PD-1)^9,10^, and other subset of cells can be measured in peripheral blood of patients with BC^2,5,11–13^. However, the function of systemic immune cells in the peripheral blood of BC patients remains relatively unexplored.

MDSCs constitute a population of immature myeloid cells with an extensive variety of immunosuppressive properties^14^. Their role in cancer has been elucidated, demonstrating in different studies that higher levels of MDSCs represent an adverse prognostic factor^15–17^. The main mechanisms for immunosuppression that MDSCs set up are the following: alteration of L-arginine metabolism through Arginase-1 (Arg-1) production and via iNOS allowing NO generation, promotion of cysteine uptake, stimulation of Tregs and tumor-associated macrophages type-2 (TAM-2) production, and inhibition of CD8 + T lymphocytes and natural killer T (NKT) function^18^_._

MDSCs can be detected in peripheral blood in cancer patients, especially in advanced stages. However, due to the intrinsic heterogeneity of these cells, analytical characterization has been challenging. At present, MDSCs are generally defined by CD45+CD3-CD19-CD20-CD56-CD16-HLADR-CD33+CD11b+expression through flow cytometry, being CD14 + and CD15 + subsets assigned as monocytic and granulocytic MDSCs, respectively^19^.

In the present work we aimed to analyze circulating MDSCs, Tregs and activated (OX40+) and inhibited (PD-1+) T lymphocyte cells from ABC patients treated with 1st line treatment for advanced disease and comparing the immune profile evolution with two groups of response, clinical benefit (CB) versus progression of disease (PD). In addition, this immune profile before starting treatment was also compared with a healthy women (HW) cohort. The results obtained support the hypothesis that consider these immune cells as promising predictive response biomarkers in ABC, useful in monitoring response to treatment and, even, as potential therapeutic targets.

## Materials and methods

Patients consecutively diagnosed of ABC and HW were invited to participate in the study at Virgen Macarena University Hospital (VMUH) in Seville (Spain). Protocol was approved by the VMUH´s institutional review board (ref. LCM-INM-2015–01/Law 14/2007, of July 3) according to the ethical principles included in Declaration of Helsinki 1964 (2013 update)^20^. Research was partially supported by a grant from the Andalusian Public Foundation Progress and Health (PI-0502-2014 FPS-2014). All the patients gave written informed consent to participate in this study.

Patients received systemic therapies following local protocols based on International Guidelines (NCCN^21^ and ABC Guidelines^22^). Clinical response was assigned following Response Evaluation Criteria in Solid Tumors version 1.1 (RECIST v 1.1.)^23^ considering Clinical Benefit (CB) the sum of Complete Responses (CR), Partial Responses (PR) and Stabilization of Disease (SD). ABC patients were classified in luminal A/B, triple negative and HER2 + subtypes, by immunohistochemistry criteria according to the established St Gallen international guidelines^24^.

### Flow cytometric analyses in whole blood samples

**Figure 1:**
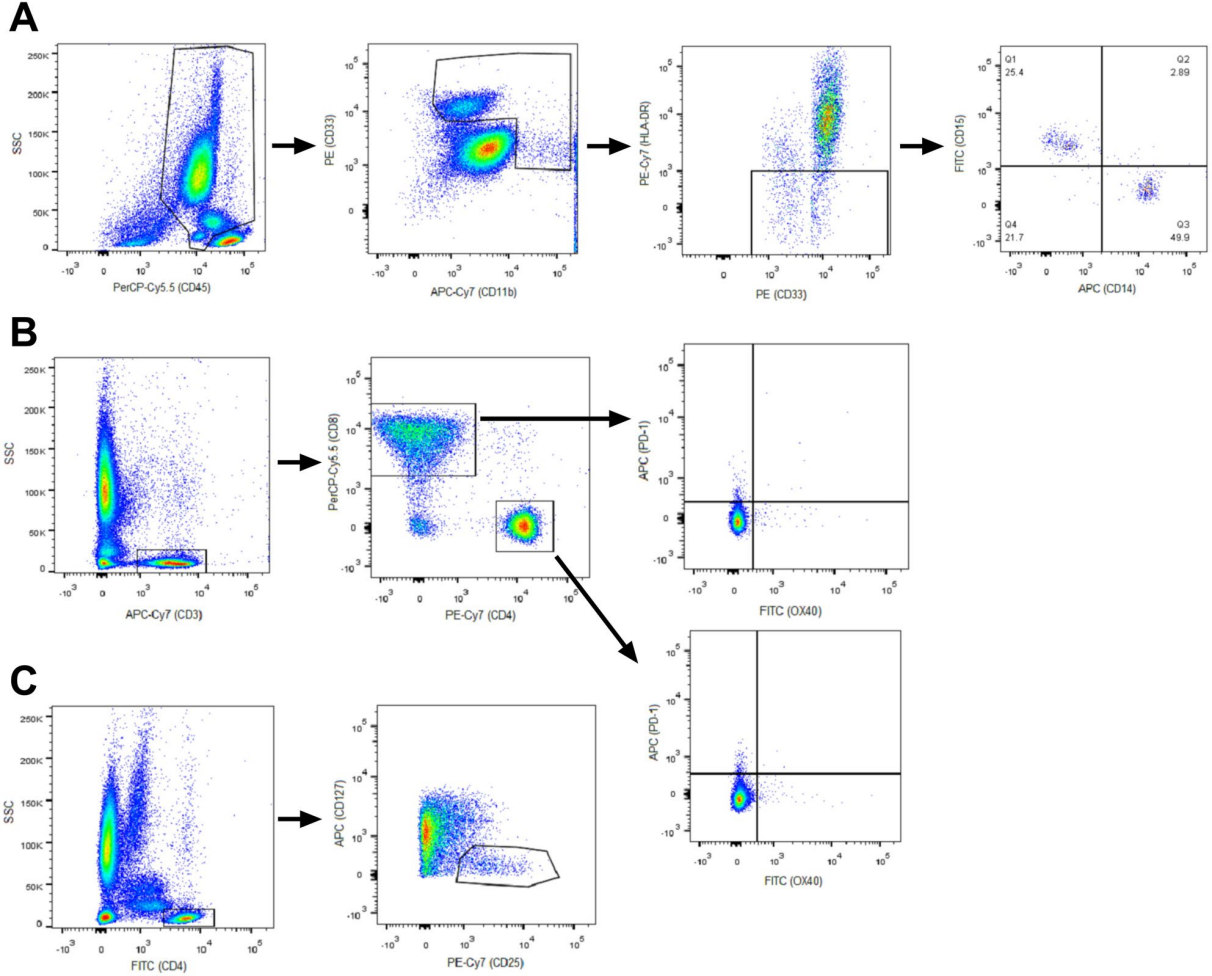
Gates of cell populations carried out by flow cytometry. M-MDSCs and G-MDSCs (**A**), CD4 + and CD8 + from OX40 + PD-1- and PD-1 + OX40- T cells (**B**), regulatory T cells (**C**).

Peripheral blood samples were collected from subjects in EDTA-K3 tubes before treatment onset and pre-dose to cycle 3 and cycle 6, to determine MDSCs, Tregs, and OX40 + and PD-1 + T lymphocytes (TL) counts. Cell populations were determined by flow cytometry of whole blood using the BD FACSCanto II flow cytometry system from EDTA-K3 tubes and gated as shown in Fig. 1. M-MDSCs were determined as CD45+CD11b+CD33+HLA-DR-CD14+CD15-, G-MDSC as CD45+CD11b+CD33+HLA-DR-CD14-CD15+, Tregs as CD3+CD4+CD25+CD127-, activated TL were determined as CD3+CD4+OX40+TL and CD3+CD8+OX-40+TL, inhibited TL as CD3+CD4+OX40-PD-1+and CD3+CD8+OX40-PD-1+. The absolute number was calculated by multiplying the percentages obtained from flow cytometry with total leukocyte and lymphocyte count obtained from hematologic count (Sysmex CS-1000). Total MDSCs were calculated as the sum of M-MDSC and G-MDSC counts, total activated T cells as the sum of CD3+CD4+OX40+PD1- and CD3+CD8+OX40+PD1- T cells counts, and total inhibited T cells as the sum of CD3+CD4+PD1+OX40- and CD3+CD8+PD1+OX40- T cells counts (Fig. 1).

### Monoclonal antibodies

Antibodies were obtained from Becton Dickinson Immunocytometry Systems (BDIS, San Jose, CA, USA) and were used at the manufacturer’s recommended concentrations.

MDSCs: PerCP-Cy5.5 Mouse Anti-Human CD 45 (ref no. 564105), APC-Cy7 Rat Anti-CD11b (ref no. 557657), PE Mouse Anti-Human CD 33 (ref no. 555450), PE-Cy7 Mouse Anti-Human HLA-DR (ref no. 560651), FITC Mouse Anti-Human CD 14 (ref no. 555397) and APC Mouse Anti-Human CD 15 (ref no. 551376).

Treg kit (ref no. 560249), including FITC anti-Human CD4, PE-Cy7 anti-Human CD25, and Alexa-Fluor 647 anti-Human CD127.

Activated and inhibited T Lymphocytes: FITC Mouse Anti-Human OX-40 (CD134) ref no. 555837, PE Mouse Anti-Human CTLA-4 (CD152) (ref no.555853), PerCP-Cy5.5 Mouse Anti-Human CD8 (ref no. 565310), PE-Cy7 Mouse Anti-Human CD4 (ref no. 557852), APC Mouse Anti-Human PD-1 (CD279) (ref no. 558694), APC-H7 Mouse Anti-Human CD3 ref no. 560176).

### Data analysis

Statistical analysis was performed by SPSS 26.0 software package (SPSS Inc., Chicago, IL, USA). Normal distribution of analyzed variables was checked by watching histogram, box plot, Q-Q plot- and the outcomes of normality tests of Kolmogorov–Smirnov (samples ≥ 50) and Shapiro–Wilk (samples < 50). Basal lymphocytes distributions from ABC patients versus HW were compared using non-parametric U-Mann–Whitney test. P value of lymphocytes distributions before, C3 and C6 of systemic therapies and from CB vs PD were obtained using Friedman test and adjusted using the Bonferroni multiple testing correction method. Correlations among cells populations were carried out using Spearman correlation coefficient. Statistically significant differences were considered at the 95% level of confidence (p ≤ 0.05).

## Results

**Figure 2:**
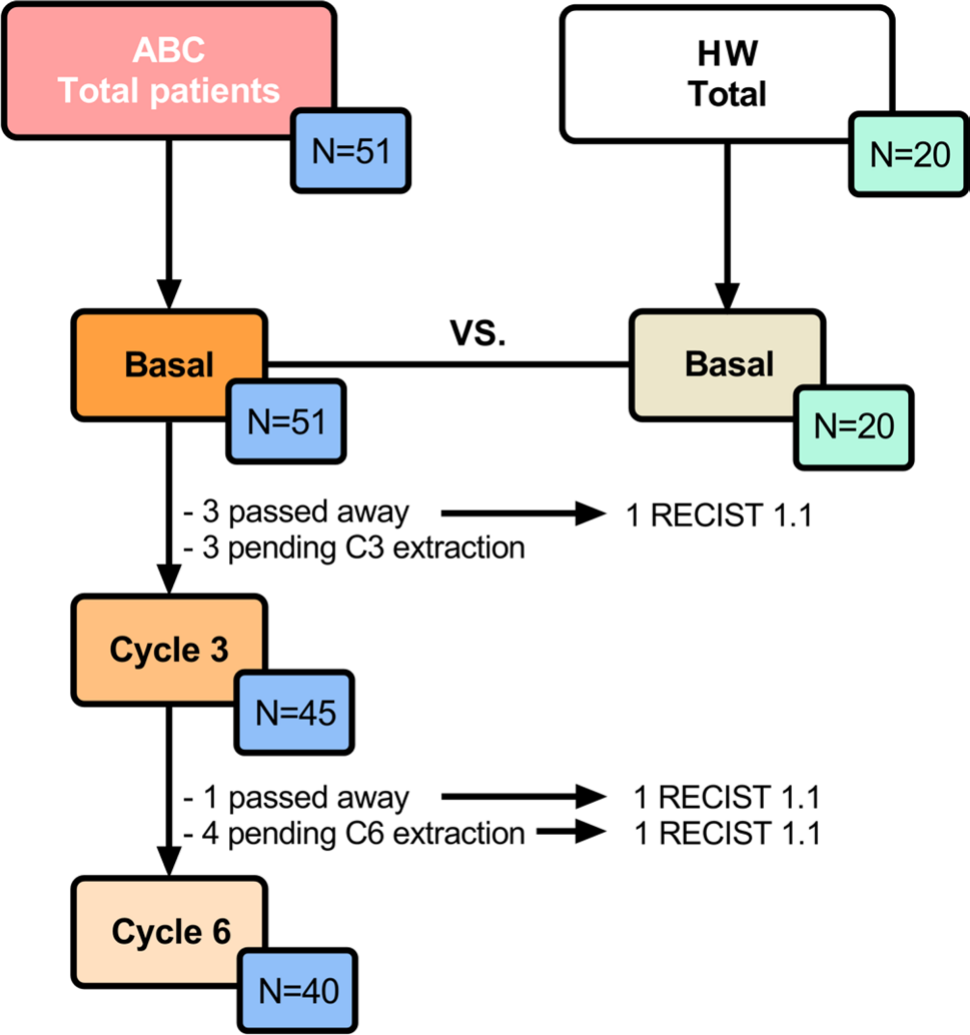
Diagram. HW, healthy women; ABC, advanced breast cancer; RECIST v1.1, response evaluation criteria in solid tumors.

From 15 January 2016 to 13 February 2020 a total of 20 HW and 51 ABC patients signed informed consent and were consecutively recruited to participate in the present study. Main clinico-pathological characteristics can be summarized as follows: most of the patients (82%) were included in the luminal breast cancer entities. All patients (100%) were diagnosed of advanced disease, and were included prior to any antineoplastic therapy in this setting. 33% had liver disease, 18% had lung metastasis and 77% had bone infiltration. Patients received 1st line treatment for ABC following local protocols based on International Guidelines and according to the tumor subtype based on the expression of RE/RP and HER2. 61% were treated with cyclin-dependent kinase 4/6 inhibitors plus hormonotherapy, 18% with hormonotherapy alone, 16% received chemotherapy, 6% were treated with anti-her2 monoclonal antibodies and 2% with chemoimmunotherapy. By 13 February 2020, the following basal (before starting treatment) measurements from peripheral blood were taken: CD8+T and CD4+T lymphocytes with expression of OX40, CD8+T and CD4+T lymphocytes with expression of PD-1, Tregs and MDSC levels. Furthermore, in ABC patients these levels were determined approximately 3 months after starting treatment (cycle 3, C3) and approximately 6 months after starting treatment (cycle 6, C6) to monitor their evolution. In C3, measurements were undertaken in 45 patients since three of them died due to PD; in two patients, response status could not be established by RECIST 1.1 criteria, and finally in three women C3 extraction had not yet be done due to SARS-CoV-2 (COVID-19) pandemic. C6 analysis could be performed in 40 patients since one of them passed away due to clinical PD and in another four patients last extraction had not yet be done either for the same reason as in C3. Objective response by RECIST 1.1 criteria could be established in 48 patients after using computed tomography (CT) in C3 (+ /− 30 days) Fig. 2.

### Clinical significance in ABC of circulating MDSC

####  MDSC levels are increased in ABC patients

Baseline MDSC levels before starting treatment in ABC patients (median cells/μl, 35.43; interquartile range (IR) = 180.73) were higher than in HW (median, 17.53; IR = 16.96) (p = 0.001).

**Figure 3:**
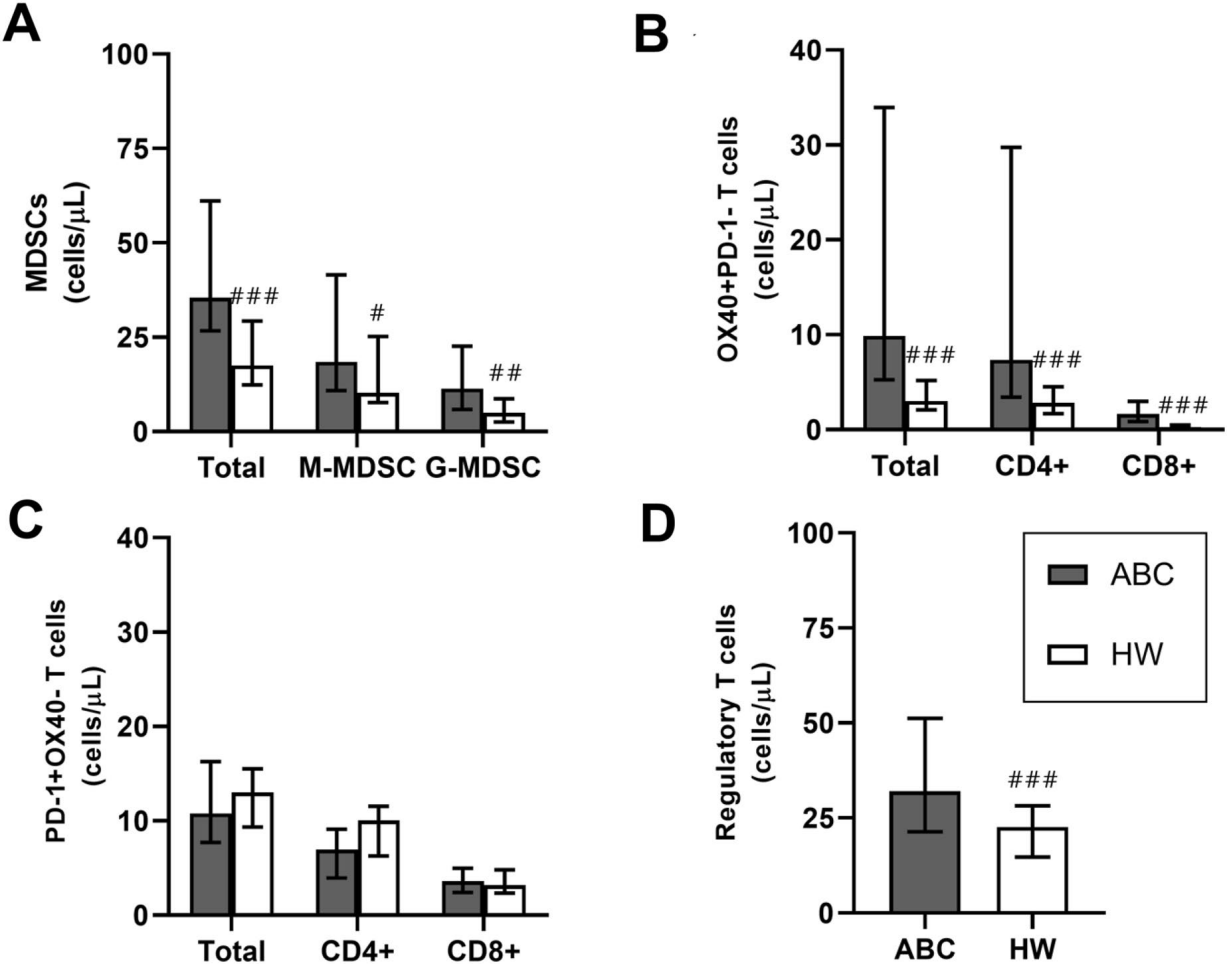
Basal medians of MDSCs, OX40 + , PD-1 + and regulatory T lymphocytes levels (cells/ul) in ABC patients and HW. *MDSCs, myeloid derived suppressor cells; M-MDSC, monocytic MDSCs; G-MDSC, granulocytic MDSCs; ABC, advanced breast carcinoma; HW, healthy women.* #, ## and ###, p ≤ 0.05, p ≤ 0.01 and p ≤ 0.001 compared with ABC patients. *Different scales are used*.

In addition, basal levels of granulocytic and monocytic MDSCs were also significantly higher in ABC patients compared to HW (p ≤ 0.035). Medians were 18.46 (IR = 30.67) cells/μl in ABC vs 10.28 (IR = 17.48) cells/μl in HW for M-MDSC and 11.38 (IR = 57.60) cells/μl in ABC vs 4.99 (IR = 16.96) cells/μl in HW for G-MDSC. Table S1; Fig. 3.

#### Low circulating MDSC levels are associated with clinical benefit

MDSC, M-MDSC and G-MDSC levels decreased along treatment in ABC patients with CB whilst they increased in those with PD, being the difference statistically significant (p ≤ 0.004) in ABC patients with CB vs PD at C6.

**Figure 4:**
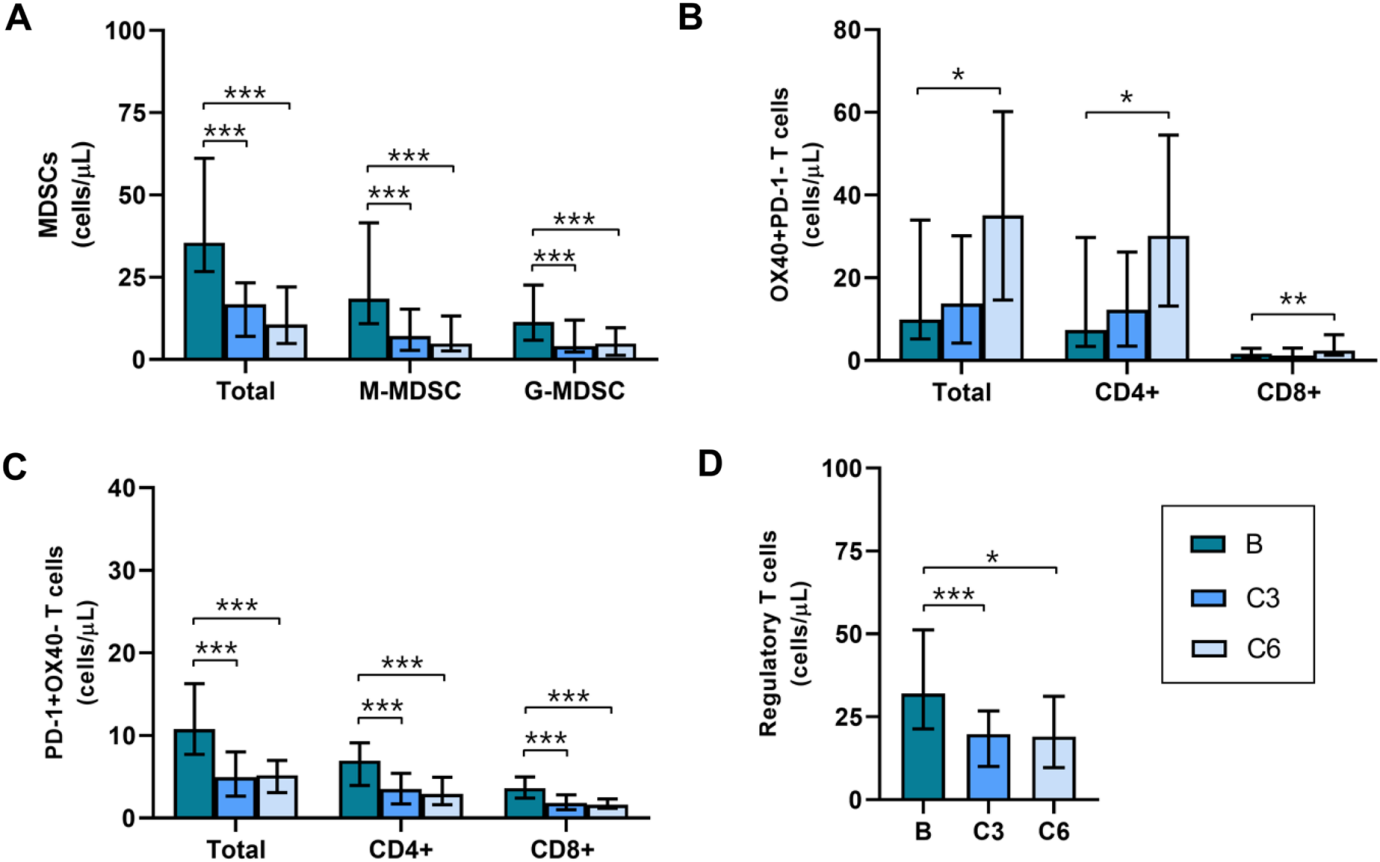
Basal, C3 and C6 medians of MDSCs (**A**), OX-40+ (**B**), PD-1+ (**C**) and regulatory T (D) lymphocytes levels (cells/ul) in ABC patients. *MDSCs, myeloid derived suppressor cells; M-MDSC, monocytic MDSCs; G-MDSC, granulocytic MDSCs; Tregs, regulatory T lymphocytes; C3, cycle 3; C6, cycle 6.* *, ** and ***, p ≤ 0.05, p ≤ 0.01 and p ≤ 0.001 compared with basal measurements, respectively. *Different scales are used*.

**Figure 5:**
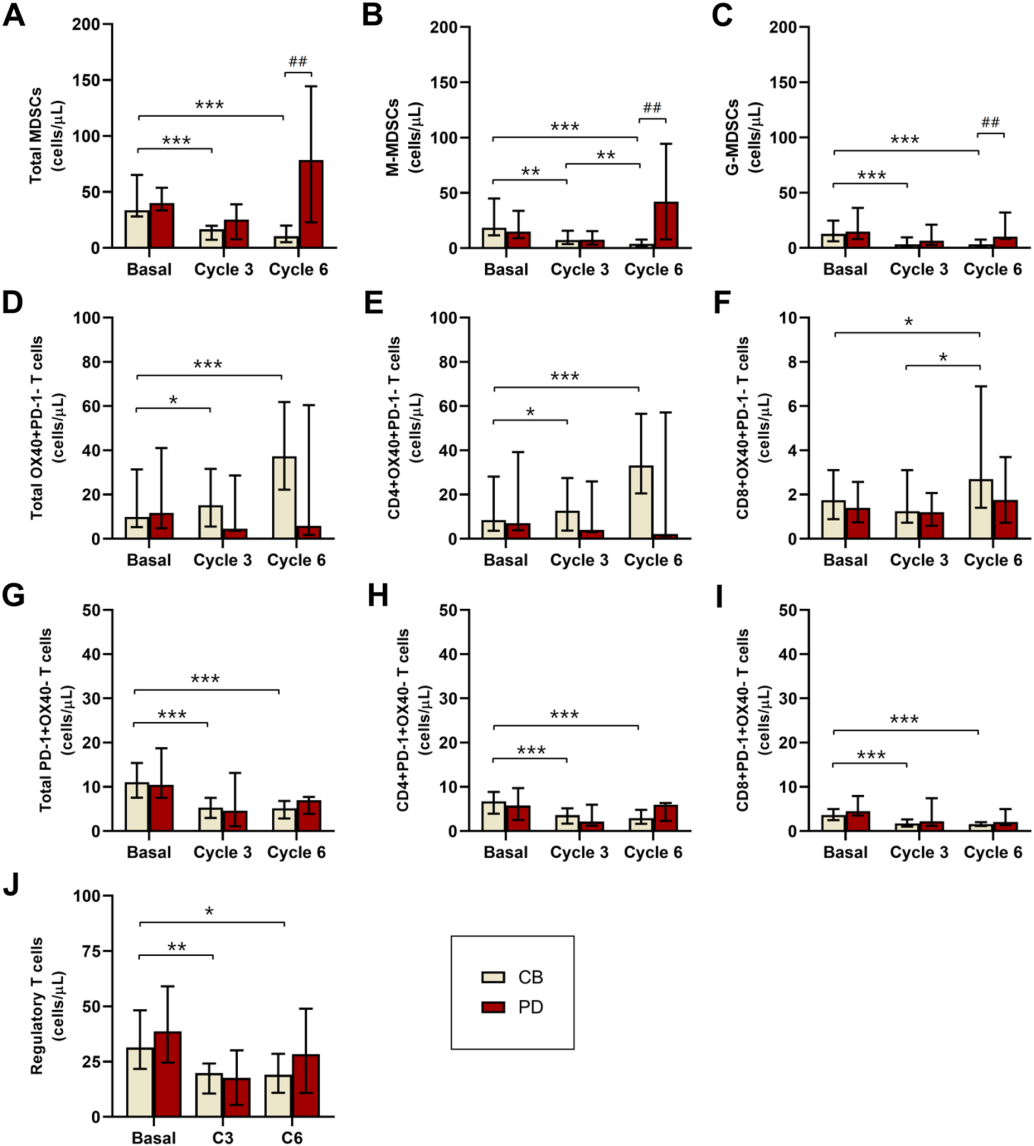
Basal, C3 and C6 medians of MDSCs (**A**, **B**, **C**), OX-40+ (**D**, **E**, **F**), PD-1 + (**G**, **H**, **D**) and regulatory T (**J**) lymphocytes levels (cells/ul) in ABC patients according to response. *MDSCs, myeloid derived suppressor cells; M-MDSC, monocytic MDSCs; G-MDSC, granulocytic MDSCs; Tregs, regulatory T lymphocytes; CB, clinical benefit; PD, progression of disease; C3, cycle 3; C6, cycle 6.* *, ** and ***, p ≤ 0.05, p ≤ 0.01 and p ≤ 0.001 compared with basal measurements, respectively. ##, p ≤ 0.01 compared with CB patients. *Different scales are used*.

**Figure 6:**
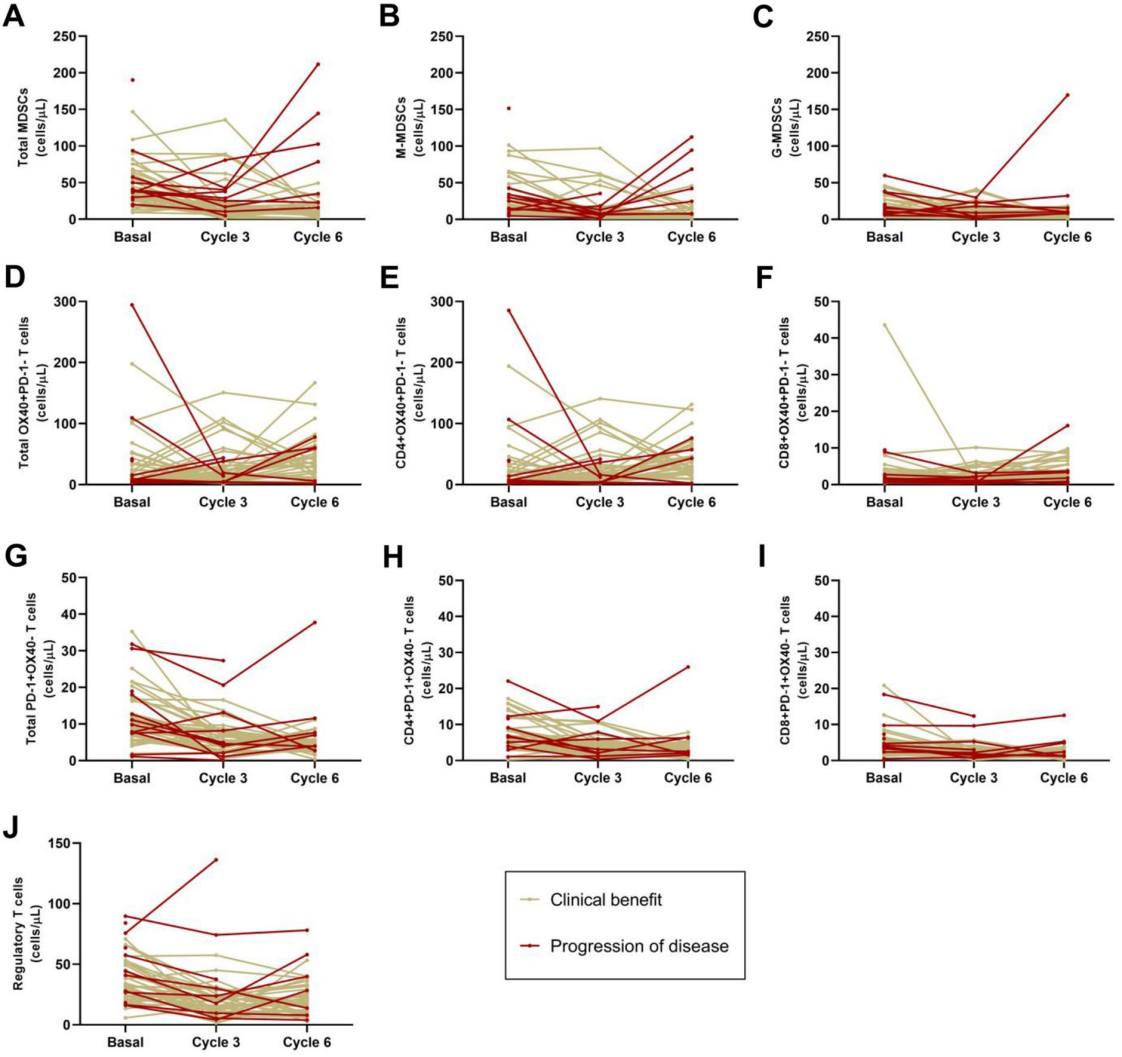
The dynamic response for each patient. In particular, each line represents the response trend from basal to C6. Red and yellow lines represent patients with progression of disease and clinical benefit, respectively. *MDSCs, myeloid derived suppressor cells; M-MDSC, monocytic MDSCs; G-MDSC, granulocytic MDSCs*.

Medians of MDSC were 33.63 (IR = 39.34) at baseline vs 10.36 (IR = 15.84) cells/μl at C6 in ABC patients with CB while they were 37.48 (IR = 20.63) at baseline vs 78.54 (IR = 128.63) cells/μl at C6 in ABC patients with PD. Medians of M-MDSC were 18.46 (IR = 30.67) at baseline vs 4.15 (IR = 6.48) cells/μl at C6 in ABC patients with CB while were 16.20 (IR = 22.97) at baseline vs 42.04 (IR = 86.99) cells/μl at C6 in ABC patients with PD. Medians of G-MDSC were 12.74 (IR = 18.14) at baseline vs 3.36 (IR = 7.03) cells/μl at C6 in ABC patients with CB while they were 14.20 (IR = 28.56) at baseline vs 10.00 (IR = 24.23) cells/μl at C6 in ABC patients with PD. Tables S2-S3; Figs. 4, 5 and 6.

### OX40 expression from CD4+ T and CD8+ T lymphocytes in ABC

#### Increased OX40+ T lymphocyte levels are detected in ABC patients

Basal total OX-40+, CD4+ T and CD8+ T lymphocyte levels before starting treatment in ABC patients were higher than in HW. Medians of total OX40+ T lymphocyte were 9.89 (IR = 28.71) cells/μl in ABC patients vs 3.01 (IR = 3.01) cells/μl in HW, being the difference statistically significant (p < 0.001). Table S1; Fig. 3.

#### Increased OX40 + T lymphocyte levels in ABC patients are correlated with clinical benefit

OX-40+, CD4+ OX40+ and CD8+ OX40+ T levels increase along treatment in ABC patients with CB while decrease in those with PD. Values at baseline vs C6 showed statistically significant differences (p ≤ 0.05) in ABC patients with CB but not in PD.

Medians of OX40 + T lymphocyte levels in ABC patients with CB were 9.89 (IR = 28.64) at baseline vs 37.28 (IR = 43.54) cells/μl at C6; CD4 + OX40 + T were 8.32 (IR = 25.46) at baseline vs 33.22 (IR = 40.49) cells/μl at C6, and CD8 + OX40 + T were 1.85 (IR = 2.37) at baseline vs 2.63 (IR = 5.72) cells/μl at C6. Tables S2-S3; Figs. 4, 5 and 6.

### PD-1 expression from CD4 + T and CD8 + T lymphocytes in ABC

#### PD-1+ T lymphocyte levels decrease in ABC patients

Basal total PD-1+ T and CD4+  PD-1+ T lymphocyte levels before starting treatment in ABC patients were slightly lower than in HW, but differences did not reach statistical significance. Medians of total PD-1+ T lymphocytes were 10.77 (IR = 8.55) cells/μl in ABC patients vs 13.00 (IR = 6.19) cells/μl in HW, (p = 0.245). Table S1; Fig. 3.

#### Decreased PD-1+ T lymphocyte levels in ABC patients associate clinical benefit

PD-1+, CD4+ PD-1+ and CD8+ PD-1+  T levels diminished along treatment in ABC patients regardless of response, however values at baseline vs C6 showed statistically significant differences (p ≤ 0.001) only in ABC patients with CB but not in PD. Medians of PD-1+ T lymphocyte levels in ABC patients with CB were 11.16 (IR = 8.99) at baseline vs 4.82 (IR = 3.74) cells/μl at C6; CD4+ PD-1+ T were 6.95 (IR = 5.08) at baseline vs 2.93 (IR = 3.12) cells/μl at C6, and CD8+ OX-40+ T were 3.62 (IR = 2.45) at baseline vs 1.51 (IR = 5.52) cells/μl at C6. Tables S2–S3; Figs. 4, 5 and 6.

### Levels of regulatory T cells (Tregs) in ABC

#### Tregs levels are increased in ABC patients

Basal Tregs levels before starting treatment in ABC patients were higher than in HW with a median of 32.05 (IR = 29.84) vs 22.61 (IR = 13.57) cells/μl, being the difference statistically significant (p = 0.001). Table S1; Fig. 3.

#### Decreased Tregs levels are observed in ABC patients with clinical benefit

Tregs levels decreased along treatment in ABC patients, being the difference statistically significant (p = 0.008) in ABC patients with CB.

Medians of Tregs were 31.87 (IR = 27.87) at baseline vs 19.01 (IR = 16.64) cells/μl at C6 in ABC patients with CB while they were 39.30 (IR = 52.24) at baseline vs 28.36 (IR = 32.03) cells/μl at C6 in ABC patients with PD. Tables S2-S3; Figs. 4, 5 and 6.

### Correlation among the different immune markers

Positive Spearman correlations between Tregs and MDSCs (r_s_ = 0.30; p < 0.001), PD-1 + OX40- T cells and Tregs (r_s_ = 0.51; p < 0.001), and between PD-1 + OX40- T cells (r_s_ = 0.35; p < 0.001) and MDSCs were observed, while the correlation was negative between CD4 + OX40 + PD-1- T cells and MDSCs (r_s_ = -−0.17; p = 0.043). Table S4.

### Clinical risk variables for immune markers

Visceral disease was associated with an increased risk of having lower basal levels of CD8 + OX40 + T lymphocytes relative to non-visceral disease (OR 10.82, 95% IC 1.35–86.91), being statistically significant (p = 0.025). Visceral disease was also related with lower total OX40 + T lymphocyte levels at baseline (OR 6.39, 95% IC 0.98–41.45) and lower CD4 + OX40 + T lymphocytes in cycle 6 (OR 6.28, 95% IC 0.99–39.95), but not statistically significant (p 0.052). Progression free interval < 24 months was related with higher Treg (OR 25.43, 95% IC 0.89–727.50) and lower CD8 + OX40 + T (OR 38.20, 95% IC 0.69–2099.65) levels in cycle 6 relative to progression free interval > 24 months, but not statistically significant (p 0.059 and p 0.075, respectively). Overall survival was related with lower CD4 + OX40 + T (OR 26.79, 95% IC 0.77–932.59) levels in cycle 6, but not statistically significant (p 0.069). Others clinical variables such as breast cancer subtype (luminal vs. TNBC vs. HER2), age at diagnosis and type of therapy received (hormonotherapy vs. chemotherapy vs. cyclin-dependent kinase 4/6 inhibitor vs. anti-her2 monoclonal antibodies) were also analyzed but they were not associated with an increased risk of having lower or higher levels of the different immune markers studied. Table S5.

## Discussion

In the last few years, growing translational evidence has established immune cells from tumoral microenvironment (TME) as a prominent hallmark in BC that should not be overlooked^25–30^. Although the interactions between the different immune components of TME in tissue are complex, and most of the mechanisms, still unknown, some functions of immune cells are being deciphered, not only in tissue but also in peripheral blood where the functions of these cell populations are less explored.

Relationship between the presence of TILs and MDSCs in TME and the response to treatment and survival of patients has been described. Thus, in certain subtypes of breast cancer, i.e. triple-negative and HER2 breast subtypes, a higher presence of TILs in TME implies improved results in pathological complete responses in the neoadjuvant setting and an impact in survival is suggested, although evidence at this point is more debatable^26,31^. A relation between the presence of elevated circulating MDSCs and prognosis have been established, being associated with advanced cancer stage, higher tumor burden, lower progression-free survival (PFS) and overall survival (OS) rates^17,32–35^ as well as with the response to cancer treatment by decreasing the efficacy of chemotherapy, radiotherapy, immunotherapy or other targeted therapies^36–38^.

MDSCs represent a heterogeneous population of immature myeloid cells among which we can distinguish two main types based on their phenotype and morphology: the granulocytic subtype (G-MDSC) and the monocytic subtype (M-MDSC). In our research, we have used the following surface markers to identify them: CD45 + CD11b + CD33 + HLA-DRlow/ − in addition to CD14-CD15 + for G-MDSCs and CD14 + CD15- for M-MDSCs^17^. Slightly higher levels of M-MDSCs over G-MDSCs have been observed in our patients. M-MDSC is a subtype that seems to be related with a greater immunosuppressive role, through NO generation, in comparison with G-MDSC^39^_._

In physiological conditions, the granulocyte–macrophage colony-stimulating factor (GM-CSF) induces myelopoiesis while the granulocyte colony-stimulating factor (G-CSF) and the macrophage colony-stimulating factor (M-CSF) stimulate the differentiation of granulocytes and macrophages in response to pathogen-derived signals like Toll-like receptors (TLR) ligands, damage-associated molecular patterns (DAMPs) and pathogen-associated molecular pattern (PAMPs) molecules. These events trigger the mobilization of neutrophils and monocytes with the production of inflammatory cytokines and costimulatory molecules. In pathological conditions of chronic inflammation or cancer, the myelopoiesis continues and is characterized, among other events, by the overproduction of MDSCs^40^. Firstly, the expansion phase of immature myeloid cells takes place through the influence of factors produced by the tumor stroma or the bone marrow like GM-CSF, G-CSF, M-CSF, stem cell factor (S-SCF) or the vascular endothelial growth factor (VEGF). Secondly, the suppressing action of MDSCs is executed by the production of reactive oxygen species (ROS), cytokines (TGF-β, IL-10, IL-6), the expression of enzymes such as indoleamine 2,3-dioxygenase (IDO) or arginase-1 (ARG1) and the production of prostaglandin E2 (PGE2)^41,42^.

At the TME level, tumors that induce expansion of MDSCs in their surrounding stroma contribute to the tumor-induced immune suppression mechanisms by inhibiting the effector function of T cells and natural killer cells through several cellular exhaustion and inactivation mechanisms^43^_,_ which are enhanced by the increased production of Tregs. The generation of ROS impair recognition through the class II major histocompatibility complex (MHC II) while nitric oxide (NO) inhibits the IL-2 receptor (IL-2R) that, in turn, affects negatively signaling via T cell receptor (TCR). Furthermore, there is a depletion of L-arginine due to the expression of high levels of ARG1 which leads to a decrease of the average life of mRNA in the CD3ζ chain of T cells. Aforementioned events, added to decreased secretion of IFN-γ and granzyme B, damage the activity of effector T lymphocytes, and may even lead to their apoptosis^44^_._

There is an immunosuppressive role of MDSCs and Tregs with influence in the anergy of T cells in the tumoral microenvironment^45^. There may also be a greater expression of immune checkpoint molecules like PD-1/PDL-1 to damage or delay the immune response by inhibiting T cells or the expression of immune system co-stimulators such as OX40. These biomarkers seem potential promissory targets for promotion of immunotherapy and personalized therapy^27^. Furthermore, some new emerging data suggest that the presence of IDO and ARG-1 by MDSCs upregulates the expression of PD-1 over MDSCs, that is, by blocking PDL-1 we would manage to mitigate the activity of MDSCs^46^.

In this work, positive Spearman correlations between circulating Tregs and MDSCs, PD-1+ OX40- T cells and Tregs, and between PD1+ OX40- T cells and MDSCs were observed, while the correlation between OX40+ PD-1- T cells and MDSCs was inverse, suggesting interrelationships between the different cell populations as previously mentioned.

We also studied the different clinical risk variables for immune markers, but only visceral disease was statistically significant associated with an increased risk of having lower basal levels of CD8+ OX40+ T lymphocytes relative to non-visceral disease. The small sample size did not allow to obtain more conclusive results with the rest of the clinical variables.

The studies in peripheral blood that correlate the levels of the different cell populations with the diagnosis of cancer or even with the response to treatment are scarce. Almand B et al.^47^ and Markowitz J et al.^15^, observed not only that the levels of MDSCs in peripheral blood were associated with tumor burden of ABC patients, but also that the decrease of MDSC levels improved the therapeutic results. Following this observation, Díaz-Montero et al.^17^ and Bergenfelz et al.^5^ also reported an increase in M-MDSCs in patients with BC, and that these levels were higher in metastatic disease, compared to early disease and healthy cohort. Furthermore, Bergenfelz et al.^5^ correlates high levels of M-MDSCs with suppressed T-cell function in patients. Our research group already revealed the immunosuppressive role of Tregs in a study with patients diagnosed with early breast cancer under neoadjuvant treatment. In that study, patients had higher levels of Tregs than healthy ones^12^. These results have also been reported by authors such as Liyanage UK et al.^48^ and Bates GJ et al.^49^, highlighting that higher levels of Tregs were associated with worse prognostic factors such as high tumor grade, greater lymph node involvement and disease burden. Tregs inhibition was correlated with a reduction of the primary tumor and metastases^50^.

In our research, ABC patients showed increased levels of MDSCs and Tregs in peripheral blood in comparison with healthy women. Therefore, it is plausible to consider that immune profile of the patients is impaired, facilitating an immune microenvironment that favors the tumor development and progression. Patients that received cancer treatment and experienced clinical benefit, experienced a drop in MDSCs and Tregs levels, whilst those patients in which disease progressed showed an increase in MDSCs levels. These findings suggest that this could be a reversible process, as immune profiling of the patients improve when disease back to be under control.

Regarding the expression of OX40, the author Hamidinia M et al.^51^ observed that CD4 + T lymphocytes expressing OX40 exerted antitumor activity and a better prognosis in patients with BC. Fu Y et al.^52^ announced that the immunosuppressive action of Tregs could be reversed through an increase in the expression of OX40.

PD-1 / PD-L1 inhibitory pathway can be used to silence the immune system by increasing the expression of PD-L1 on the tumor cell surface with binding to PD-1 of T lymphocytes. In breast cancer, this axis has prognostic and predictive implications, especially in triple negative subtype^53–55^. Further, PD-1/PD-L1 is the target of antitumoral treatments after the survival results of the phase III study IMpassion130 / NCT02425891^27^ that supported the accelerated approval by the FDA of atezolizumab in combination with nabpaclitaxel for triple negative ABC with PD-L1 expression.

In our work, we could demonstrate enhanced levels of CD4+ and CD8+ T lymphocytes with expression of OX40 in ABC patients compared to healthy women. However, it was in the healthy cohort where we observed a higher percentage of T lymphocytes with expression of the co-repressive molecule PD-1, which reveal the great complexity of the mechanisms of interrelation among the immune system elements beyond those described here: the release of inhibitory cytokines, the expansion of T regulators and tumor-associated macrophages (TAM), the expression of immune checkpoint and the presence of MDSCs working all together to create this immunosuppressive microenvironment^2,18,37–39^. Nonetheless, with respect to CD4+ T and CD8+ T expressing OX40, we could observe a rise in these cells in the ABC cohort that reached CB. On the contrary, patients that progressed showed declining and lower levels of OX40 T cells. Furthermore, a significant decrease in CD4+ T and CD8+ T with expression of PD-1 along treatment in ABC patients with CB could be ascertained.

These findings appear promising, but further knowledge about the immunosuppressive role of MDSCs is required, as well as the co-repressive / costimulatory activity of PD-1 / OX40 molecules, respectively. The depletion in the levels of MDSCs, Tregs and the increase of OX40 along antineoplastic treatment, whether this is hormonotherapy alone, chemotherapy, hormonotherapy plus cyclin-dependent kinase inhibitors 4/6, or other combined strategies, could improve immune profile of the patients reverting their immunosuppressive status with respect to cancer cells. These determinations could also become potential biomarkers to monitor in a simple way by means of a simple peripheral blood extraction the response to treatment.

Several combinations of treatments are being carried out in preclinical studies that target MDSCs and immune checkpoint, boosting the immune effector activity in cancer. Recently, it has been revealed that the inhibition of PI3K, JAK/STAT and CDK4/6 pathways improve the infiltration of cytotoxic CD8+ T type TILs in mouse models with triple-negative breast cancer. This effect seems tightly related to the decrease of the MDSCs levels, what ultimately facilitate the action of effector T cells. In top of that, this effect may be boosted by blocking some specific immune checkpoint inhibitors^56^. Consequently, targeting MDSCs aimed to deplete them can potentially increase the efficacy of the modern immune checkpoint inhibitors currently in use for several oncological diseases^57^.

Results of this study put the focus on potential biomarkers easily detectable in peripheral blood to monitor the response of antineoplastic treatments in ABC. Likewise, it permits foresee new targets for immunomodulatory strategies in breast cancer. There are uncountable therapeutic possibilities at this point, since there are different options to boost the immune response of the host whether employing classic agents (doxorubicin, cyclophosphamide, gemcitabine, and so on) or newer agents such as anti-PD-1 (antibodies that block the inhibiting and proapoptotic effect of their signals in T cells), anti-OX40 or anti-CD40 (antibodies that stimulate the activating signaling of T lymphocytes) and/or within the context of combinations.

Despite of the small sample size, the data of this study are promising as statistically significant differences are found between ABC patients vs healthy cohort and during the monitoring of the different subgroups of immune cells throughout treatment according to the response achieved. The results obtained support the hypothesis that consider these immune cells as promising predictive response biomarkers in ABC, useful in monitoring response to treatment and, even, as potential therapeutic targets. Nevertheless, additional studies with higher sample size are required to confirm these results. New avenues of research can be suggested from these findings since a sort of immune liquid biopsy might proportionate extraordinarily valuable and complementary information in BC, not only in the advanced setting but even also during follow-up of BC patients previously treated with curative purposes.

### Ethics approval

Protocol was approved by the VMUH´s institutional review board (ref. LCM-INM-2015–01/Law 14/2007, of July 3) according to the ethical principles included in Declaration of Helsinki 1964 (2013 update).

### Consent to participate

All the patients signed written informed consent to participate in this study.

### Consent for publication

All the authors gave their consent to submit this version of the manuscript.

## Supplementary Information

Supplementary Information.

## Data Availability

Data and materials are accessible under formal request.
