# Publisher Correction: Circulating immune biomarkers in peripheral blood correlate with clinical outcomes in advanced breast cancer

**DOI:** 10.1038/s41598-021-96901-8

**Published:** 2021-08-30

**Authors:** Natalia Palazón‑Carrión, Carlos Jiménez‑Cortegana, M. Luisa Sánchez‑León, Fernando Henao‑Carrasco, Esteban Nogales‑Fernández, Massimo Chiesa, Rosalía Caballero, Federico Rojo, María‑Adoración Nieto‑García, Víctor Sánchez‑Margalet, Luis de la Cruz‑Merino, the Spanish Breast Cancer Group (GEICAM) and the Spanish Group for Immunobiotherapy of Cancer (GÉTICA)

**Affiliations:** 1 Clinical Oncology Department, Virgen Macarena University Hospital, Seville, Spain; 2 Department of Medical Biochemistry and Molecular Biology, and Immunology, School of Medicine, Virgen Macarena University Hospital, University of Seville, Seville, Spain; 3 GEICAM (Spanish Breast Cancer Research Group), Madrid, Spain; 4 Pathology Department, IIS-Fundación Jimenez Diaz-CIBERONC, Madrid, Spain; 5 Department of Preventive Medicine and Public Health, Faculty of Medicine, University of Seville, Seville, Spain; 6 Medicine Department, University of Seville, Seville, Spain

Correction to: *Scientific Reports* 10.1038/s41598-021-93838-w, published online 13 July 2021

In the original version of this Article, Víctor Sánchez-Margalet was omitted as a corresponding author. Correspondence and requests for materials should also be addressed to margalet@us.es.

The original Article has been corrected.

