# Supplementary material for: Circulating immune biomarkers in peripheral blood correlate with clinical outcomes in advanced breast cancer
Source: Sci Rep. 2021 Jul 13;11:14426. doi: 10.1038/s41598-021-93838-w (PMC8277895; doi:10.1038/s41598-021-93838-w)
Supplement: Supplementary file 1 — Supplementary Information. [file 41598_2021_93838_MOESM1_ESM.pdf]

SUPPLEMENTARY

MDSCs	Total	M-MDSCs	G-MDSCs
**ABC**	35.43 (180.73)	18.46 (30.67)	11.38 (57.60)
**HW**	17.53 (16.96)	10.28 (17.48)	4.99 (16.96)
**P value**	0.001$^{\#\#\#\#}$	0.035$^{\#\#}$	0.004$^{\#\#\#}$
**OX40+PD-1- T cells**	**Total**	**CD4+**	**CD8+**
**ABC**	9.89 (28.71)	7.36 (26.33)	1.64 (2.14)
**HW**	3.01 (3.10)	2.81 (2.83)	0.28 (0.26)
**P value**	<0.001$^{\#\#\#}$	<0.001$^{\#\#\#}$	<0.001$^{\#\#\#}$
**PD-1+OX40- T cells**	**Total**	**CD4+**	**CD8+**
**ABC**	10.77 (8.55)	6.95 (5.14)	3.63 (2.57)
**HW**	13.00 (6.19)	10.04 (5.27)	3.18 (2.47)
**P value**	0.245	0.156	0.591
**Regulatory T cells**			
**ABC**	32.05 (29.84)		
**HW**	22.61 (13.57)		
**P value**	0.001$^{\#\#\#}$		

Table S1. Basal MDSC, OX40+PD-1-, PD-1+OX40- and regulatory T cell levels in ABC patients vs. HW. Cell levels (cells/uL) are median and interquartile range. P values were obtained using Mann-Whitney U test. *MDSCs, myeloid derived suppressor cells; M-MDSCs, monocytic MDSCs; G-MDSCs, granulocytic MDSCs; ABC, advanced breast carcinoma; HW, healthy women.*

MDSCs	Total	M-MDSCs	G-MDSCs
**Basal**	35.43 (180.73)	18.46 (30.67)	11.38 (57.60)
**Cycle 3**	16.80 (16.75)	7.09 (12.54)	4.03 (9.64)
**Cycle 6**	10.61 (21.51)	4.87 (10.64)	4.86 (8.31)
**P value**	<0.001***	<0.001***	<0.001***
	**B-C3**: <0.001***<br>**B-C6**: <0.001***<br>**C3-C6**: 0.841	**B-C3**: 0.001***<br>**B-C6**: <0.001***<br>**C3-C6**: 0.539	**B-C3**: 0.001***<br>**B-C6**: <0.001***<br>**C3-C6**: 0.735
**OX40+PD-1- T cells**	**Total**	**CD4+**	**CD8+**
**Basal**	9.89 (28.71)	7.36 (26.33)	1.64 (2.14)
**Cycle 3**	13.75 (25.94)	12.23 (22.72)	1.24 (2.28)
**Cycle 6**	35.10 (45.56)	30.17 (41.34)	2.46 (4.97)
**P value**	0.045*	0.025*	0.007*
	**B-C3**: 0.343<br>**B-C6**: 0.042*<br>**C3-C6**: 0.327	**B-C3**: 0.281<br>**B-C6**: 0.022*<br>**C3-C6**: 0.943	**B-C3**: 0.837<br>**B-C6**: 0.006*<br>**C3-C6**: 0.101
**PD-1+OX40- T cells**	**Total**	**CD4+**	**CD8+**
**Basal**	10.77 (8.55)	6.95 (5.14)	3.62 (2.56)
**Cycle 3**	5.02 (5.01)	3.51 (3.69)	1.82 (1.81)
**Cycle 6**	5.17 (3.90)	2.93 (3.33)	1.61 (1.12)
**P value**	<0.001***	<0.001***	<0.001***
	**B-C3**: <0.001***<br>**B-C6**: <0.001***<br>**C3-C6**: 0.801	**B-C3**: <0.001***<br>**B-C6**: <0.001***<br>**C3-C6**: 0.723	**B-C3**: <0.001***<br>**B-C6**: <0.001***<br>**C3-C6**: 0.776
**Regulatory T cells**			
**Basal**	35.05 (29.84)		
**Cycle 3**	19.79 (16.75)		
**Cycle 6**	19.01 (21.51)		
**P value**	0.001***		
	**B-C3**: 0.001***		

	**B-C6**: 0.022*<br>**C3-C6**: 0.943

Table S2. MDSC, OX40+PD-1-, PD-1+OX40- and regulatory T cell levels in ABC patients during basal, cycle 3 and cycle 6. Cell levels (cells/uL) are median and interquartile range. P values were obtained using Friedman test and adjusted using the Bonferroni multiple testing correction method. *MDSCs, myeloid derived suppressor cells; M-MDSCs, monocytic MDSCs; G-MDSCs, granulocytic MDSCs; B, basal; C3, cycle 3; C6, cycle 6.*

**MDSCs**	**Total**		**M-MDSCs**		**G-MDSCs**	
	**CB**	**PD**	**CB**	**PD**	**CB**	**PD**
**Basal**	33.63 (39.34)	37.48 (20.63)	18.46 (30.67)	16.20 (22.97)	12.74 (18.14)	14.20 (28.56)
**Cycle 3**	16.54 (12.88)	25.32 (31.26)	7.09 (12.54)	7.50 (12.24)	3.23 (6.70)	5.63 (20.09)
**Cycle 6**	10.36 (15.84)	78.54 (128.63)	4.15 (6.48)	42.04 (86.99)	3.36 (7.03)	10.00 (24.23)
**P values 1**	<0.001***	0.042	<0.001***	0.066	<0.001***	0.607
	**B-C3**: 0.001***<br>**B-C6**: <0.001***<br>**C3-C6**: 0.662	**B-C3**: 0.043<br>**B-C6**: 0.773<br>**C3-C6**: 0.921	**B-C3**: 0.002**<br>**B-C6**: <0.001***<br>**C3-C6**: 0.006**	-	**B-C3**: <0.001***<br>**B-C6**: <0.001***<br>**C3-C6**: 0.382	-
**P values 2**	**Basal:** 0.550<br>**C3:** 0.375<br>**C6:** 0.003$^{\#\#}$		**Basal:** 0.654<br>**C3:** 0.765<br>**C6:** 0.004$^{\#\#}$		**Basal:** 0.489<br>**C3:** 0.248<br>**C6:** 0.002$^{\#\#}$	
**OX40+PD-1- T cells**	**Total**		**CD4+**		**CD8+**	
	**CB**	**PD**	**CB**	**PD**	**CB**	**PD**
**Basal**	9.89 (28.64)	11.71 (36.90)	8.32 (25.46)	6.14 (35.77)	1.85 (2.37)	1.40 (28.56)
**Cycle 3**	15.23 (26.78)	4.57 (12.90)	12.93 (25.02)	4.00 (10.97)	1.24 (2.29)	1.20 (20.09)
**Cycle 6**	37.28 (43.54)	5.91 (57.62)	33.22 (40.49)	2.22 (45.44)	2.63 (5.72)	1.75 (3.33)
**P values 1**	0.002**	0.867	0.002**	0.867	0.020*	0.163
	**B-C3:** 0.031*<br>**B-C6:** <0.001***<br>**C3-C6:** 0.162	-	**B-C3:** 0.034*<br>**B-C6:** <0.001***<br>**C3-C6:** 0.162	-	**B-C3**: 0.712<br>**B-C6**: 0.027*<br>**C3-C6**: 0.010*	-
**P values 2**	**Basal:** 0.661<br>**C3:** 0.288<br>**C6:** 0.199		**Basal:** 0.703<br>**C3:** 0.395<br>**C6:** 0.199		**Basal:** 0.686<br>**C3:** 0.630<br>**C6:** 0.419	
**PD-1+OX40- T cells**	**Total**		**CD4+**		**CD8+**	
	**CB**	**PD**	**CB**	**PD**	**CB**	**PD**
**Basal**	11.16 (8.99)	11.03 (11.18)	6.95 (5.08)	5.95 (8.58)	3.62 (2.45)	4.51 (4.34)
**Cycle 3**	5.02 (3.99)	4.96 (14.81)	3.51 (3.28)	2.16 (8.11)	1.63 (1.62)	2.24 (6.66)
**Cycle 6**	4.82 (3.74)	7.64 (4.55)	2.93 (3.12)	5.98 (4.86)	1.51 (5.72)	1.80 (3.72)
**P values 1**	<0.001***	0.651	<0.001***	0.779	<0.001*	0.091
	**B-C3**: <0.001***<br>**B-C6**: <0.001***<br>**C3-C6**: 0.902	-	**B-C3**: <0.001***<br>**B-C6**: <0.001***<br>**C3-C6**: 0.806	-	**B-C3**: <0.001***<br>**B-C6**: <0.001***<br>**C3-C6**: 0.611	-
**P values 2**	**Basal:** 0.981<br>**C3:** 0.699<br>**C6:** 0.058		**Basal:** 0.402<br>**C3:** 0.392<br>**C6:** 0.212		**Basal:** 0.686<br>**C3:** 0.630<br>**C6:** 0.240	

	**Regulatory T cells**	
	**CB**	**PD**
**Basal**	31.87 (27.87)	39.30 (52.24)
**Cycle 3**	20.79 (15.01)	17.63 (38.37)
**Cycle 6**	19.01 (16.64)	28.36 (32.03)
**P values 1**	0.008**	0.174
	**B-C3**: 0.003**<br>**B-C6**: 0.016*<br>**C3-C6**: 0.611	-
**P values 2**	**Basal:** 0.470<br>**C3:** 0.895<br>**C6:** 0.680	

Table S3. MDSC, OX40+PD-1-, PD-1+OX40- and regulatory T cell levels in ABC patients during basal, cycle 3 and cycle 6 according to response. Cell levels (cells/uL) are median and interquartile range. P values 1 were obtained using Friedman test and adjusted using the Bonferroni multiple testing correction method. P values 2 were obtained using Mann-Whitney U test. *MDSCs, myeloid derived suppressor cells; M-MDSCs, monocytic MDSCs; G-MDSCs, granulocytic MDSCs; CB, clinical benefit; PD, progression of disease; B, basal; C3, cycle 3; C6, cycle 6.*

Spearman correlation	Tregs		Total MDSCs		M-MDSCs		G-MDSCs	
	**rS**	**P value**	**rS**	**P value**	**rS**	**P value**	**rS**	**P value**
**Tregs**	-	-	0.30	<0.001	0.25	<0.001	0.35	<0.001
**Total PD-1+OX40- T cells**	0.51	<0.001	0.35	<0.001	0.33	<0.001	0.24	0.006
**CD4+PD-1+OX40- T cells**	0.52	<0.001	0.34	<0.001	0.31	<0.001	0.24	0.005
**CD8+PD-1+OX40- T cells**	0.39	<0.001	0.30	<0.001	0.30	<0.001	0.19	0.025
**Total OX40+PD-1- T cells**	-	-	-0.15	0.073	-0.19	0.026	-0.05	0.546
**CD4+OX40+PD-1- T cells**	-	-	-0.17	0.043	-0.20	0.018	-0.07	0.448
**CD8+OX40+PD-1- T cells**	-	-	-0.03	0.699	-0.03	0.683	-0.04	0.617

Table S4. Correlations among cells populations. Spearman correlation coefficient was used. *MDSCs, myeloid derived suppressor cells; M-MDSCs, monocytic MDSCs; G-MDSCs, granulocytic MDSCs; rS, Spearman correlation.*

			**Treg > median Cycle 6**		**Total OX40+ T < median Basal**		**CD4+OX40+ T < median Basal**		**CD4+OX40+ T < median Cycle 6**		**CD8+OX40+ T < median Basal**		**CD8+OX40+ T < median Cycle 6**	
Clinical variables		Ref.	P value	OR (95% IC)	P value	OR (95% IC)	P value	OR (95% IC)	P value	OR (95% IC)	P value	OR (95% IC)	P value	OR (95% IC)
**Visceral disease**	**Yes**	**No**	_	_	0.052	6.39 (0.98-41.45)	0.052	6.28 (0.99-39.95)	–	–	**0.025**	**10.82 (1.35-86.91)**	_	_
**Progression free interval**	**<24 months**	**>24 months**	0.059	25.43 (0.89-727.50)	_	_	_	_	–	–	_	_	0.075	38.20 (0.69-2099.65)
**Overall survival**	**Exitus**	**Alive**	_	_	_	_	_	_	0.069	26.79 (0.77-932.59)	_	_	_	_

Table S5. Odds ratios and clinical risk variables for immune markers. *OR, odds ratio; Ref, reference; Tregs, regulatory T lymphocytes. Only OR and P value with almost or difference statistically significant are shown.*
